# The relationship between spiritual intelligence and self-management in patients with diabetes type 1

**DOI:** 10.1186/s12902-023-01482-4

**Published:** 2023-10-23

**Authors:** Sima Rafiei, Saber Souri, Zahra Nejatifar, Mohammad Amerzadeh

**Affiliations:** 1 Social Determinants of Health Research Center, Research Institute for Prevention of Non- Communicable Diseases, Qazvin University of Medical Sciences, Qazvin, Iran; 2 Student Research Committee, School of Public Health, Qazvin University of Medical Sciences, Qazvin, Iran

**Keywords:** Diabetic mellitus, Spiritual intelligence, Self-management

## Abstract

**Background:**

Diabetes is widely recognized as one of the most pressing public health concerns globally. To manage blood glucose levels and reduce subsequent complications and mortality rates, self-management mechanisms have been found to be effective in controlling diabetes. This study aimed to investigate the association between spiritual intelligence and diabetes self-management in patients with type 1 diabetes in Qazvin, Iran.

**Methods:**

This cross-sectional study included 220 adults with type 1 diabetes aged 18–35 years who referred to an outpatient diabetes clinic of a tertiary hospital in Qazvin province, and were selected through a convenience sampling method in 2022. Two valid and reliable questionnaires were used for data collection, including the 24-item questionnaire of spiritual intelligence and self-management of type 1 diabetes for adolescents (SMOD-A). To analyze the data, correlation coefficients and multiple linear regression analysis were used.

**Results:**

The total score of spiritual intelligence was 57.24 ± 10.77, and self-management was 77.14 ± 8.92. Among different subscales of spiritual intelligence, critical thinking obtained the highest score. In self-management, the highest score was achieved for communication.Findings also revealed that spiritual intelligence could predict 7.2% of changes in self-management among diabetes patients, and its relationship with diabetes self-management was estimated at 0.27.

**Conclusion:**

The growing prevalence of diabetes worldwide underscores the significance of self-management of the disease in the well-being of patients. This study demonstrated that spiritual intelligence played a crucial role among young adults with diabetes and assisted them in coping with stressful situations. As such, placing greater emphasis on the spiritual aspects of care is necessary, especially in the healthcare of young adults who are dealing with diabetes and its complex conditions.

## Background

Diabetes is a significant health issue worldwide in the 21st century. It is a chronic metabolic and progressive illness caused by an impaired secretion of insulin or insulin function [1]. Type 1 diabetes, also known as juvenile diabetes or insulin-dependent diabetes, is a chronic condition characterized by the destruction of beta cells in the pancreas, resulting in the production of little or no insulin. Insulin is a hormone that regulates blood sugar levels by allowing glucose to enter cells for energy production [2]. One out of 300 children below the age of 18 is diagnosed with this disease, and every year, 3% is added to this number [3, 4]. Research indicates that by the year 2040, the estimated number of individuals affected by type 1 diabetes will range from 13.5 to 17.4 million. It is noteworthy that low- and lower-middle income nations will encounter the most significant proportional rise in the overall impact of this disease [5]. In Iran, a study conducted by the National Program for Prevention and Control of Diabetes revealed that 11.4% of all diabetes patients in 2016 belonged to type 1 diabetes (T1D) [6].

Self-management of T1D can be vital as a comprehensive approach to managing the disease, particularly in instances where complex decision-making is required and flexible regimens are needed. Grady and Gough have provided a definition of self-management as the continuous management of chronic conditions by individuals throughout an illness [7]. With regard to diabetes, self-management is a fundamental component of maintaining adequate blood glucose control and minimizing potential risks and mortalities among patients [8]. In self-management, both youth and their parents are responsible for engaging in various activities to address their disease, as well as fostering collaboration with healthcare providers towards the ultimate goal of managing their diabetes. [8]. Maintaining a well-regulated level of insulin, nutrition, and energy intake is essential for preventing complications related to diabetes and ensuring optimal glycemic control [9]. Various factors, including age, knowledge and awareness, communication between patients and family members, friends, and clinicians, and mental health, can significantly impact self-management among diabetic patients [10]. Spirituality has been recognized as a psychosocial factor that plays a significant role in self-management behaviors [11]. Studies have shown that individuals who possess strong spiritual beliefs and values tend to have better adaptation to disease, fewer acute episodes of sickness, and decreased complications [10, 12]. Spiritual skills are essential for enhancing problem-solving capabilities and understanding the meaning of life [13]. According to Wigglesworth, spiritual intelligence refers to the capacity to exhibit compassion and wisdom, maintain inner and outer tranquility, and disregard external circumstances and events [12]. Other studies have shown that individuals with developed spiritual intelligence are better equipped to adapt to their environment and deal more effectively with life challenges, such as periods of illness [14].

As research has consistently shown, there has been a growing interest in understanding the role of self-management in the lives of individuals with diabetes. Several studies have investigated various factors that influence self-care in these patients. For instance, Bigdely et al.‘s study in Iran revealed that several factors, including age, knowledge about the disease, good relationships between patients and physicians, and some mental conditions, significantly influence self-care among diabetic patients [12]. Other studies have shown that there is a positive correlation between spiritual beliefs, mental health, healthy hygiene behaviors, life expectancy, and self-esteem in adolescents [11, 15]. These findings suggest that healthcare providers, policymakers, and families should prioritize education and support programs to promote self-awareness and self-management skills among this group of patients.

Despite the growing body of research on the topic, there is limited information on the relationship between spirituality in type 1 diabetes patients and its role in the self-management of the disease.

Therefore, this study aimed to investigate the relationship between spiritual intelligence and self-management of the disease in T1D patients in Qazvin, Iran.

## Methods

### Study design

This cross-sectional study was conducted among type 1 diabetes patients referred to an outpatient diabetes clinic of a tertiary hospital in Qazvin province, Iran, between July and September 2022.

### Sample size

In this research, 220 patients admitted to the diabetes clinic during the study period were selected to participate in the survey using a convenience sampling method. Specifically, during the study period between July and September 2022, all patients who referred to the diabetes clinic of one of the general hospitals in Qazvin province and were available in the waiting room at the time of the researcher’s visit were invited to participate in the study. Afterward, through consideration of inclusion and exclusion criteria, a total of 220 patients who met the conditions were selected for the study. Patients aged 18 to 35 years, with a final diagnosis of diabetes, without suffering from any mental disorder, and having the literacy to read and write, participated in the study. In contrast, patients above 35 years old, those suffering from any mental disorder, taking psychiatric or narcotic drugs, unable to read and write, or disagreeing to take part in the research were excluded from the research process. After obtaining written informed consent and completing the questionnaire, patients who were conveniently accessible during the data collection period were approached by the researcher in the diabetes out-patient clinic. The researcher explained the study objectives and distributed the questionnaires among patients who were waiting for their appointments.

### Data collection

The 24-item questionnaire, developed into four subscales including critical thinking, personal meaning, transcendental consciousness, and consciousness development, was used to collect data on spiritual intelligence. The patients’ responses were scored on a 5-point Likert scale (completely false = 0, false = 1, somewhat correct = 2, correct = 3, and completely correct = 4), and the final achievable score ranged from 0 to 96. The content validity of the questionnaire was tested in multiple studies and showed satisfactory results. Moreover, its reliability was proven to be acceptable with Cronbach’s alpha of 0.90 [12]. Furthermore, to assess self-management among diabetes patients, a Self-Report Measure of Self-Management of Type 1 Diabetes for Adolescents (SMOD-A) with a 4-point Likert scale scoring method was employed. The data collection instrument comprised five subscales, including diabetes care activities, problem-solving ability, communication, parental cooperation, and goals, resulting in a final score within a range of 0 to 144. In a study by Schilling et al., the content validity of the scale was estimated at 0.93, and its reliability ranged between 0.6 and 0.88 after two weeks of use [16].

To collect data, the researcher approached young adults who referred to the diabetes out-patient clinic in a tertiary hospital in Qazvin province while they were waiting for their appointments. First, study objectives were explained to patients and after obtaining their agreement to take part in the study, the questionnaires were distributed among them.

The study procedures were approved by the Research Ethics Committee of Qazvin Medical University. The protocol contained detailed information on the ethical obligations of researchers toward participants engaging in the research project. These obligations included informed consent, privacy, anonymity, maximizing benefits, and minimizing harms, which were rigorously observed during the research process.

### Data analysis

The Statistical Package for Social Sciences (SPSS) version 20.0 (SPSS Inc., Chicago, IL, USA) was utilized for data analysis. In the first step, descriptive statistics were calculated as means and standard deviations (SD) for quantitative data and frequencies (%) for categorical variables. Then, correlation coefficients were used to assess the relationship between spiritual intelligence and self-management. Finally, multiple linear regression analysis was performed to examine the predictive role of spiritual intelligence on self-management of the disease among diabetes patients. Indeed, to mitigate confounding bias that distorts the relationship between the predicting factor and outcome variable, we first identified and measured the relationship between the predictor and one or more than one factor that might affect the occurrence of the outcome. In this study, an outcome variable was self-management and different subscales of spiritual intelligence including personal meaning, conscious development, and critical thinking were mentioned as predictors in the regression model. Age is a crucial factor that affects successful diabetes self-management in patients. Middle-aged individuals are more likely to have poor glycemic control and lower confidence in their diabetes self-management [21, 22]. Furthermore, evidence suggests that there is a statistically significant relationship between age and spiritual intelligence (p < 0.05). Older adults tend to have higher levels of spiritual intelligence and are more involved in spiritual activities. Such a sense of comfort, purpose, and meaningful life might help them pursue medication and curative regimens more strictly and therefore make more effective self-management efforts. Therefore, age needs to be adjusted for in order to accurately measure the relationship between spiritual intelligence and self-management [23]. The Statistical significance was considered at p < 0.05.

## Results

The demographic characteristics of 220 diabetes patients are presented in Table 1. Non-response during subject inclusion may also lead to sampling bias. However, in this study, the response rate was 88%, which represents an acceptable response rate. The majority of participants were male, with an average age of 26.5 ± 4.85 years and an estimated mean duration of diabetes at 4.82 ± 0.82 years. Almost 66% of participants were married, 71% had an academic university degree, and 48% had a history of diabetes in their close relatives. Furthermore, there were statistically significant relationships between age, self-management, and spiritual intelligence (p < 0.05).

**Table 1:** Socio-demographic characteristics of study participants and their spiritual intelligence

Characteristics		Frequency	%Frequency	p-value
Gender	Male	158	72	0.482
	Female	62	28	
Marital status	Married	145	66	0.066
	Single	75	34	
Educational level	Under diploma	22	10	0.052
	Diploma	41	19	
	University degree	157	71	
Duration of the disease (year)	< 5	48	22	0.164
	5–10	147	67	
	> 10	25	11	
Family history	Yes	105	48	0.113
	No	115	52	
		**Mean**	**Standard deviation**	
Age		26.5	4.85	0.047
Self-management	Diabetes care activities	16.64	4.53	0.024
	Problem-solving	13.67	7.14	
	Communication	23.12	8.42	
	Parental cooperation	22.36	5.44	
	Goals	12.78	4.95	
	Total score	77.14	8.92	

Regarding different aspects of self-management, the highest score was achieved for communication (Mean = 23.12, SD = 8.42), followed by parental cooperation (Mean = 22.36, SD = 5.44), and diabetes care activities (Mean = 16.64, SD = 4.53) (Table 1).

**Table 2:** The status of spiritual intelligence among study participants

	Dimensions	Mean	Standard deviation
**Spiritual intelligence**	Critical thinking	17.65	3.92
	Personal meaning	12.24	4.17
	Transcendental awareness	16.93	3.15
	Conscious development	12.51	3.67
	Total score	57.24	10.77

The total score of spiritual intelligence was 57.24 ± 10.77, and the score of self-management was 77.14 ± 8.92, as shown in Tables 1 and 2. The highest scores were obtained for critical thinking (Mean = 17.65, SD = 3.92), transcendental awareness (Mean = 16.93, SD = 3.15), and conscious development (Mean = 12.51, SD = 3.67) among different subscales of spiritual intelligence. Regarding different aspects of self-management, the highest score was achieved for communication (Mean = 23.12, SD = 8.42), followed by parental cooperation (Mean = 22.36, SD = 5.44), and diabetes care activities (Mean = 16.64, SD = 4.53).

The results of multiple linear regression analysis revealed that after adjusting for age, educational level, marital status, duration of the disease, and family history, spiritual intelligence was a significant predictor of diabetes self-management among patients with type 1 diabetes. Specifically, spiritual intelligence predicted 7.2% of changes in self-management among diabetes patients, and its relationship with diabetes self-management was estimated at 0.27. Additionally, examining the stepwise model, personal meaning, conscious development, and critical thinking could predict 4.8%, 4.12%, and 4.08% of diabetes self-management changes with corresponding associations with diabetes self-management (β: 0.22, 0.203, and 0.201 respectively) (Table 3).

**Table 3:** Factors associated self-management in diabetes patients

Model Summary				
Model	R	R^2^	Adjusted R^2^	SE
Total spiritual intelligence	0.27^*^	0.0729	0.0725	12.53167
Personal meaning	0.22^*^	0.0484	0.0439	12.64215
Conscious development	0.203^*^	0.0412	0.0408	11.78912
Critical thinking	0.201	0.0408	0.0403	11.86432
**Model**	**Coefficients**		**Β**	**t**
	**B**	**SE**		
Constant	62.863	4.016		13.117
Total spiritual intelligence	0.284	0.062	0.227^*^	2.96
Constant	60.147	4.298		15.446
Personal meaning	1.345	0.316	0.264^*^	4.356
Conscious development	0.419	0.116	0.247^*^	3.583
Critical thinking	0.572	0.244	0.397^*^	4.315

P < 0.05*

Adjusted for the effect of age as a confounding variable

## Discussion

This study aimed to investigate the relationship between spiritual intelligence and self-management in patients with type 1 diabetes in Qazvin, Iran. The findings showed that the mean score of spiritual intelligence was 57.24 ± 10.77, which was higher than the mean score of the instruments (46.0%). This suggests that patients with type 1 diabetes had a high level of spiritual intelligence. Another study found that critical thinking and transcendental consciousness obtained higher scores, which is consistent with the findings of this study [17]. Additionally, it is notable that 50.32% of participants had a moderate status of spiritual intelligence, which is similar to the findings of this study [18]. Furthermore, another study found that most patients (32%) had an average spiritual intelligence level, which is consistent with the findings of this study. The findings of this study are also supported by another study that showed that patients with better spiritual intelligence cope with stress more efficiently in chronic diseases and perform self-management activities better [19].

Our findings revealed statistically significant relationships between age, self-management, and spiritual intelligence (p < 0.05). Another study in Iran did not find a significant relationship between the underlying variables and the spiritual intelligence variable [17]. Our findings showed that an average score of self-management was 77.14 ± 8.92, indicating that patients with type 1 diabetes had a desirable status of diabetes self-management. Regarding different aspects of self-management, the highest score was achieved for communication, parental cooperation, and diabetes care activities. However, in other studies, the mean scores of subjects in the self-care dimension had the highest, followed by communication and collaborating with parents [8, 17].

Another study in Iran found that spiritual intelligence was not a strong predictor of changes in self-management among diabetes patients, which is consistent with the results of this study [12]. The reason for this weak prediction may be due to the nature of type 1 diabetes, which typically affects younger individuals. At this stage of life, they are still developing abstract thinking and complex issues related to values, meaning, self-awareness, and other components of spiritual intelligence. Therefore, spiritual intelligence is less emphasized in the early stages of life compared to other stages of life [13]. A similar study demonstrated a negative correlation between students’ academic achievement and spiritual intelligence. This study supports the idea that adolescents are uncertain about abstract concepts and their relationship with their regular activities, which can negatively impact their spiritual intelligence [20]. Other studies have suggested that religious beliefs can influence various aspects of people’s physical and psychological well-being and develop their skills in communication, self-confidence, and decision-making [21, 22]. However, a study on the relationship between spiritual health and control in people with type 2 diabetes did not find a relationship between health and religious activities [17].

Among different subscales of spiritual intelligence examined in a stepwise model, personal meaning, conscious development, and critical thinking could predict 4.8%, 4.12%, and 4.08% of diabetes self-management changes with corresponding impact on diabetes self-management. A study conducted in Iran also revealed a significant relationship between spiritual intelligence and desirable psychological sense. In this study, patients with a better score of spiritual intelligence could bear more problems. It also found that spiritual intelligence can lead to immaterial, non-compulsory aspects and improves day-to-day performance and health [23]. Another study on patients with multiple sclerosis in the form of spiritual intelligence training sessions found that training in spiritual intelligence assisted patients in all aspects of adjusting to sickness and maintaining their life satisfaction [24].

### Strengths and limitations

Our study is the first of its kind conducted in Iran regarding self-management in patients with diabetes. However, there were some limitations to the study. Firstly, there were not many specific centers for diabetes in Qazvin, which limited our study sample size. A convenience sampling method in this study led to conclusions that were likely biased based on the accessibility of study participants and therefore limited study generalizability to the population from which the sample was selected conveniently. Secondly, the study used questionnaires to collect data, which, due to relying on structured and predefined response options, might restrict participants’ ability to provide in-depth responses. Also, in this approach, there is no possibility of explaining questions and clarifying them for participants, which might lead to some biases. Thirdly, the study was cross-sectional, which means that the study variables were only measured once, making it difficult to establish a cause-and-effect relationship between them. Furthermore, the study used convenience sampling, which might have resulted in biased conclusions due to the availability of participants. To address these limitations, the study emphasized the representativeness of the source population rather than computing a deductive sample size. Furthermore, the inclusion criteria were established, and efforts were made to recruit patients who met the determined conditions.

## Conclusion

The increasing prevalence of diabetes in both the world and Iran underscores the importance of self-management in the lives of individuals with diabetes. Several studies have sought to improve the health outcomes of diabetic patients by enhancing their spiritual intelligence. This study demonstrates that spiritual intelligence is positively correlated with self-management in individuals with diabetes and their overall health status. Therefore, healthcare managers, policymakers, and families should prioritize education programs to promote religious attitudes and self-intelligence among patients with diabetes, enabling them to better manage their condition and lead a more fulfilling life.

## Data Availability

The datasets used and/or analyzed during the current study available from the corresponding author on reasonable request. The entire dataset is in Farsi language. The Data can be available in English language for the readers and make available from the corresponding author on reasonable request.
